# Oscillatory activity in auditory cortex reflects the perceptual level of audio-tactile integration

**DOI:** 10.1038/srep33693

**Published:** 2016-09-20

**Authors:** Michael Plöchl, Jeremy Gaston, Tim Mermagen, Peter König, W. David Hairston

**Affiliations:** 1 Institute of Cognitive Science, University of Osnabrück, Albrechtstraße 28, 49069 Osnabrück, Germany; 2 Human Research and Engineering Directorate, Army Research Laboratory, Aberdeen Proving Ground, MD, USA; 3 Department of Neurophysiology and Pathophysiology, University Medical Center Hamburg-Eppendorf, Martinistr. 52, 20246 Hamburg, Germany

## Abstract

Cross-modal interactions between sensory channels have been shown to depend on both the spatial disparity and the perceptual similarity between the presented stimuli. Here we investigate the behavioral and neural integration of auditory and tactile stimulus pairs at different levels of spatial disparity. Additionally, we modulated the amplitudes of both stimuli in either a coherent or non-coherent manner. We found that both auditory and tactile localization performance was biased towards the stimulus in the respective other modality. This bias linearly increases with stimulus disparity and is more pronounced for coherently modulated stimulus pairs. Analyses of electroencephalographic (EEG) activity at temporal–cortical sources revealed enhanced event-related potentials (ERPs) as well as decreased alpha and beta power during bimodal as compared to unimodal stimulation. However, while the observed ERP differences are similar for all stimulus combinations, the extent of oscillatory desynchronization varies with stimulus disparity. Moreover, when both stimuli were subjectively perceived as originating from the same direction, the reduction in alpha and beta power was significantly stronger. These observations suggest that in the EEG the level of perceptual integration is mainly reflected by changes in ongoing oscillatory activity.

The mechanisms underlying cross-modal integration are frequently studied by means of visual-auditory localization paradigms, where vision usually acts as the dominant sense which influences auditory perception^1^,^2^,^3^,^4^,^5^,^6^,^7^. This is in line with the more general notion that the sense that provides the highest perceptual accuracy regarding the task at hand usually biases the estimates in modalities with lower accuracy^2^,^8^,^9^,^10^,^11^,^12^,^13^,^14^,^15^. Moreover, the direction and magnitude of this bias has been shown to depend on the temporal and spatial disparity between the presented cues and on their perceived causal relation^3^,^4^,^5^,^6^,^7^,^16^.

However, in how far these observations also hold for audio-tactile cue combinations is not resolved yet. As both modalities possess similar localization accuracies^17^,^18^, they are likely to exert a mutual rather than a uni-directional localization bias. Earlier experiments have already demonstrated that simultaneous audio-tactile stimulation can indeed introduce localization biases^13^,^14^,^15^. But to what extent the cross-modal influences are mutual and to what degree they depend on the spatial disparity and perceptual interrelation between the presented stimuli has – to our knowledge – not been systematically studied yet.

Furthermore, audio-tactile integration has been related to neural activity in auditory cortex, usually by comparing blood oxygenation level dependent responses (BOLD) or event-related potentials (ERPs) during passive bimodal stimulation with the summed activity during unimodal auditory and tactile stimulation, respectively^5^,^19^,^20^,^21^,^22^,^23^,^24^,^25^,^26^. However, in paradigms that do not involve an active task, it is not possible to determine whether and to what degree the observed neural interactions also reflect perceptual integration. Even in studies that do demonstrate that changes of neural activity are accompanied by changes in task performance^27^, it is not clear whether these effects arise from perceiving the presented cues as causally interrelated (i.e. emanating from the same source) or from more general bottom-up interactions (e.g. a partial integration of the cues).

Here, we investigate the integration of simultaneously presented auditory and tactile stimuli in a novel 360° localization paradigm. Moreover, we manipulated the likelihood of perceiving both stimuli as being interrelated by presenting them with either similar or dissimilar amplitude modulation. As our participants reported the location of both the auditory and the tactile cue, our paradigm enables us to simultaneously determine cross-modal interactions in both modalities and thus to differentiate between different levels of audio-tactile integration on a trial-by-trial basis.

In addition to our participants’ behavioral performance we also investigated their EEG activity emerging from temporal cortical areas. By analyzing both neural oscillations and ERPs we aim to shed light at their respective roles in the neural integration of auditory and tactile signals.

## Methods

### Participants

We recorded behavioral responses and EEG from 14 participants (7 female/7 male, age range 18–56 years) during an audio–tactile localization task. All experimental procedures were approved by the US Army Research Laboratory Internal Review Board in accordance with the Army Human Research Protection Office and conformed to the Declaration of Helsinki and national guidelines. All subjects participated voluntarily without financial compensation, and were informed about the experimental procedure, possible risks or discomforts, and their right to abort the experiment at any time without having to state a reason. After signing informed consent, each participant was screened for normal hearing [better than 25 dB (HL)] through air conduction at octave intervals in the 250 Hz–4000 Hz frequency range.

### Stimuli

**Figure 1:**
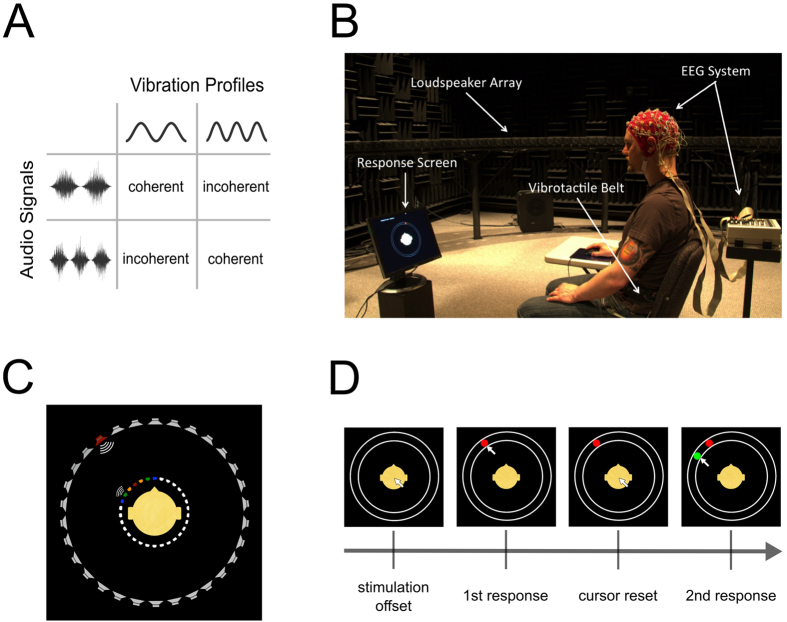
Stimuli and experimental paradigm: (**A**) Amplitude profiles of auditory and tactile stimuli and their possible combinations. (**B**) Experimental setup. (**C**) Stimulus locations and disparities. In the shown example, the auditory cue (outer circle, red) is presented at −36°. Possible locations for the simultaneously presented tactile cue in this case are indicated by the colored symbols of the inner circle (with the active vibromotor shown at −24° disparity relative to the auditory stimulus). (**D**) The participants indicated their localization estimates by subsequently adjusting a red dot (auditory) and a green dot (tactile) on a continuous circle on the screen (or vice versa). Between the first and the second response, the cursor was reset to the center of the screen.

Auditory stimuli were created in MATLAB (The MathWorks, Inc.). Each stimulus had a duration of one second and consisted of white noise that was amplitude modulated with an either 2 Hz or 3 Hz sinusoidal window (cf. Fig. 1A), thus giving rise to the perception of either two or three smooth bursts of white noise within one second. The stimuli were presented over an array of calibrated high quality loudspeakers (described below) at an average sound pressure level of 74 dB at the listening location.

Tactile stimulation was provided using a vibromotor belt developed at the University of Osnabrück (http://feelspace.cogsci.uni-osnabrueck.de, see also refs 28 and 29). The belt contains 30 vibromotors (Precision Microdrives Ltd., Model 310–101, typical normalized amplitude: 1.4 G) that are equidistantly spaced in 12° intervals and individually controlled via MATLAB and a USB connection. Like the auditory stimuli, each tactile stimulus had a duration of one second with either a 2 Hz or 3 Hz amplitude modulation. Thus, the vibration profiles corresponded to the amplitude envelopes of the 2 Hz and 3 Hz modulated auditory stimuli (Fig. 1A).

To ensure that auditory and tactile stimuli were precisely synchronized, we calibrated their timing by simultaneously recording the sound and vibration onset with two microphones, one placed at the listening location and the other on top of the active vibromotor, respectively. Subsequently, we adjusted both stimulus onsets such that they occurred within a time window of <10 ms, thus accounting for any delays emerging from the audio system, the distance from the sound source to the listener, and the response latencies of the vibromotors.

### Experimental procedure

The experiment was conducted at the Environment for Auditory Research facility at the US Army Research laboratory, which is a hemi-anechoic room with a ring of loudspeakers (Meyer Sound MM-4XP) arranged around the listener (for a detailed description of the research space, see ref. 30). More specifically, the participants were seated in the center of a circular array of 30 loudspeakers, each separated by 12°, and wore a belt with 30 evenly spaced vibromotors, each of which matched the direction of one of the loudspeakers (Fig. 1B,C). Throughout the experiment a screen in front of the participant displayed a simple head shape surrounded by a ring thus illustrating the participant’s position with respect to the loudspeaker array (cf. Fig. 1D). Auditory and tactile cues were presented either individually (unimodal condition) to provide a baseline for localization performance or simultaneously (bimodal condition) with varying amounts of spatial disparity (i.e., either from the same direction or with an offset of 12°, 24°, or 36°; cf. Fig. 1C). Additionally, on bimodal presentation trials, the modulation frequencies of both stimuli (2 Hz or 3 Hz) were matched in a way that they were either coherent (e.g., both 2 Hz) or non-coherent (e.g., auditory 2 Hz, tactile 3 Hz, Fig. 1A) in order to facilitate or hamper the perception that both stimuli are interrelated. After each trial, a cursor appeared on the response screen and the participants were asked to report the perceived location of the presented auditory and/or tactile cue(s) via mouse click(s) on the displayed ring. More specifically, the participants had to move the cursor from the center of the screen toward the ring. As soon as they reached the ring’s outer rim, a colored dot (red for responses to auditory stimuli, green for tactile stimuli) appeared; it could then be adjusted along the ring and confirmed by pressing the mouse button. In the bimodal condition, because both locations of the auditory and tactile cues had to be reported, the response to the modality that was reported first remained visible on the screen, and the cursor moved back to the center of the screen. Subsequently, they had to follow the same procedure to localize the second modality (Fig. 1D). After the participants’ response(s) the cursor disappeared and after a 500 ms delay the next trial started.

All participants performed two experimental blocks, one in which they first localized the auditory cue and then the tactile cue, and another in which the response order was reversed. Both blocks were randomly interspersed with unimodal trials, and the block order (i.e., auditory response first or tactile response first) was counterbalanced across subjects.

Each subject performed a total of 1080 trials consisting of 2 blocks × 30 stimulus locations × 9 stimulus combinations (unimodal auditory, unimodal tactile and bimodal with seven different disparities: −36°, −24°, −12°, 0°, +12°, +24°, and +36° where −/+ denote an offset to the left and right, respectively) × 2 levels of stimulus coherence (coherent or non-coherent).

### EEG recording and preprocessing

EEG data were recorded using a BioSemi ActiveTwo amplifier (BioSemi, Amsterdam, Netherlands) at a sampling rate of 512 Hz. Sixty-four pre-amplified surface electrodes were placed on the scalp according to the standardized international 10–20 electrode placement system^31^. Furthermore, we used two electrodes attached to the lateral orbital rim of the left and the right eye and two electrodes attached superior and inferior to the right eye in order to monitor horizontal (HEOG) and vertical (VEOG) eye movements, respectively.

For preprocessing, EEG data were imported into EEGLAB^32^ and filtered between 2 Hz and 100 Hz, re-referenced to the average activity of all electrodes, and cut into epochs ranging from −2 s to 2 s relative to stimulation onset. The 60 Hz line noise was removed using Cleanline (https://www.nitrc.org/projects/cleanline/), and after visual inspection, trials containing high amplitude noise and other easily identifiable confounds such as sudden electrode drifts and jumps were discarded. To account for the rank reduction of the data due to average referencing, we removed the scalp electrode located at the inion (Iz) leaving us 63 scalp and two EOG channels for further analysis. Subsequently we performed Independent Component Analysis^33^ (ICA) to decompose each participant’s data into 65 maximally statistically independent signal sources (ICs). Finally, we estimated the equivalent dipole location of each IC within an MNI (Montreal Neurological Institute) standard brain using the DIPFIT function as implemented in EEGLAB^32^.

For further analysis we only kept those ICs that were located in either left or right auditory cortical areas and responded to auditory stimulation. Note that on the scalp level auditory cortical activity that emerges from both left and right cortices simultaneously may peak at more central electrode locations. In this case, however, dipole localization remains ambiguous depending on whether choosing one or two dipoles for the localization procedure. As a result the corresponding ICs may be confused with other more centrally located sources and consequently we did not include such ICs in our analysis. Restricting our selection to the above-mentioned criteria we found a total of 16 ICs (8 in left and 8 in right auditory cortex) originating from 8 of our 14 participants. All analyses were performed in IC space, that is, on the respective activations of each individual IC and without back-projecting them to sensor space.

### EEG data analysis

The preprocessed EEG data were further analyzed in Fieldtrip^34^. IC activation data were baseline corrected on each trial to the interval between −300 ms and 0 ms with respect to stimulus onset before computing the event-related potential (ERP). To investigate the time course of spectral power in each IC, we computed the respective time-frequency representations (TFRs), with each segmented into 250 ms-long overlapping data windows, advancing in 5-ms steps from −250 ms to +1500 ms. We analyzed the frequency range between 4 Hz and 30 Hz by multiplying each data segment with a Hanning window and then computing the fast Fourier transform (FFT). We also analyzed spectral power in the gamma frequency band (30 Hz–100 Hz) but this time using a multitaper approach^35^,^36^ instead of a Hanning window. However, because our analysis did not yield any significant differences or systematic patterns in the gamma range, the respective TFRs are not shown or discussed in the following. Finally, in order to obtain power changes with respect to baseline, all time-frequency bins in a given IC were normalized to the average pre-stimulus power in each frequency.

### Statistics

Behavioral performance was evaluated using t-tests or, in those cases where two factors and their interactions were tested simultaneously (e.g., uni-/bimodal stimulation and stimulus location or stimulus disparity and stimulus coherence), by means of two-way repeated measures ANOVAs, which we computed separately for each modality. Furthermore, we used simple correlation to investigate linear trends (indicated by the correlation coefficient r). In the EEG analysis, statistical significance for both ERPs and TFRs was computed over ICs by means of a cluster-based non-parametric permutation test^37^. Here, ERP or TFR differences that exceed a predefined threshold (two standard deviations from the mean in this case) are clustered and summed across adjacent time points and, for time-frequency analysis, across frequency bins. By randomly exchanging conditions in a random subset of subjects (in our case, ICs) before averaging and clustering, an alternative observation is obtained. Repeating this procedure multiple times (here: n = 1000) yields a reference distribution under the null hypothesis, which is then used to test how often clusters of the observed size are expected to randomly occur, while at the same time accounting for multiple comparisons over time samples and/or time-frequency bins.

## Results

### Localization performance and cross-modal bias

We first assessed our participants’ auditory and tactile localization performance in the unimodal and bimodal conditions at 0° stimulus disparity (i.e., spatially congruent multisensory signals). Scores were calculated based on the angular differences between the participants’ responses and the actual stimulus locations in each trial (single-trial errors). As a measure for general localization accuracy, we then computed the absolute error, which corresponds to the average magnitude (i.e., absolute values) of single-trial errors.

**Figure 2:**
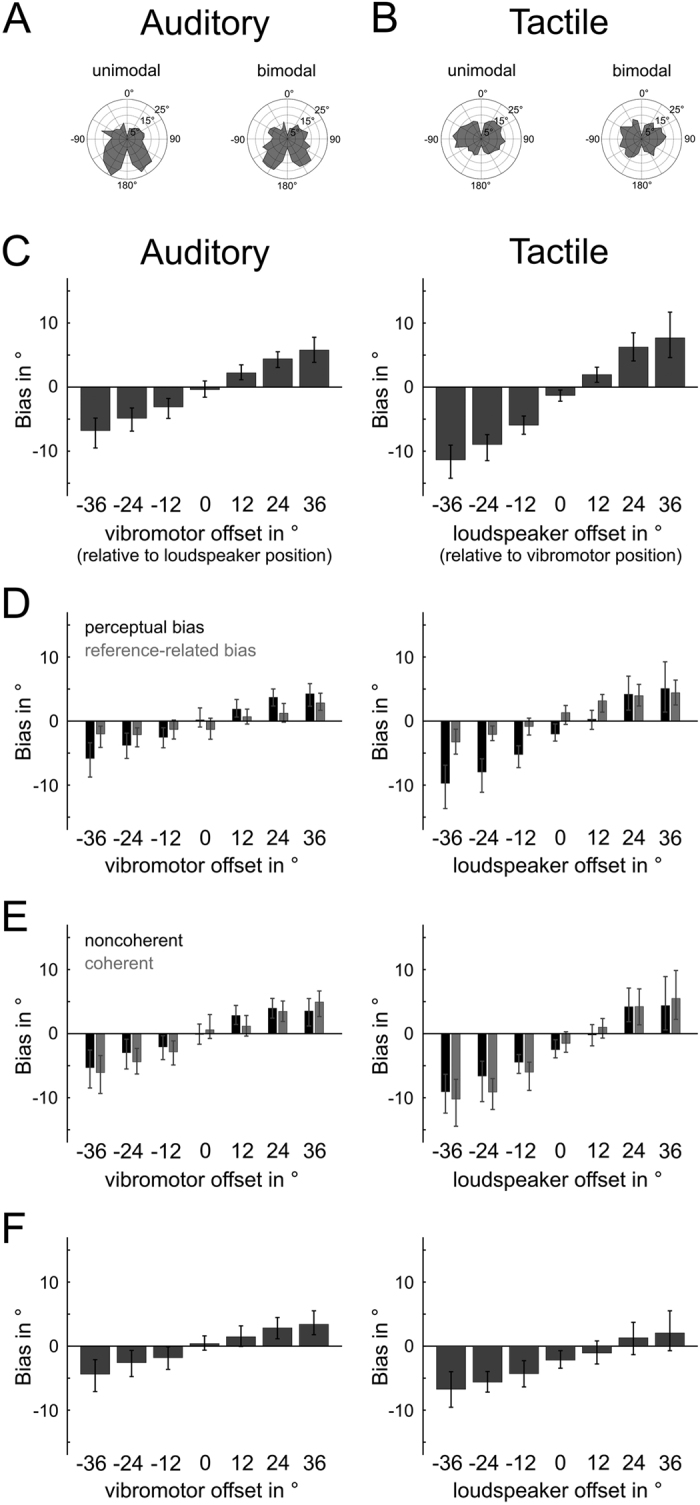
Behavioral results: (**A**) Average magnitudes of auditory localization errors with respect to stimulation angle. (**B**) Average magnitude of tactile localization errors with respect to stimulation angle. (**C**) Systematic localization biases as a function of stimulus disparity (negative = left, positive = right). The error bars indicate the 95% confidence intervals at each disparity. (**D**) Comparison between the portion of the observed bias that arises from cross-modal interactions (“perceptual bias,” represented in black and estimated in trials where the modality of interest was localized first) and the portion which may arise from a possible bias towards the first response (i.e., “reference-related bias,” represented in gray and defined as the difference between trials in which the respective modality was localized second and the perceptual bias). (**E**) Systematic localization biases broken into trials with non-coherently (black) and coherently (gray) modulated stimulus pairs (cf. Fig. 1A). (**F**) Partial integration: Systematic localization biases after excluding fully integrated trials (i.e., trials in which both modalities were localized within ±6° relative to each other).

In the unimodal conditions, we found the overall absolute (i.e. unsigned) error to be 12.8° in the auditory and 11.7° in the tactile modality. Both the magnitudes and the distributions of auditory and tactile localization errors (Fig. 2A,B) were consistent with previous reports^17^,^18^. A two-way repeated measures ANOVA revealed no significant differences of overall errors between the two modalities (F(29, 377) = 1.39, p = 0.26). However, as expected from the error distributions across angles (Fig. 2A,B), the errors significantly depended on stimulus location (F(29, 377) = 8.88, p < 0.001). Additionally, a significant interaction between angle-specific errors and modality indicates that the respective error distributions differ between auditory and tactile localization performance.

In the spatially congruent bimodal condition, the absolute errors were slightly, but non-significantly reduced to 12.5° and 11.3°, respectively. As in the unimodal conditions before, the ANOVA confirmed that stimulus angle influenced localization performance (F(29, 377) = 7.90, p < 0.001) and that the respective error distributions across angles differed between modalities (F(29, 377) = 7.22, p < 0.001). Again there was no significant difference between the two modalities, although this time the respective p-value only marginally exceeded the critical level of 0.05 (F(29, 377) = 4.31, p = 0.058).

We furthermore tested whether the participants’ responses to auditory or tactile stimuli were biased towards one particular side of the body. Therefore, we computed the overall systematic (i.e. signed) error by taking the mean of signed single-trial errors across all stimulus angles. As a result, any significant deviations from 0° would indicate a systematic bias toward the left (negative values) or right (positive values) body side. While not significant in the auditory modality (unimodal: +0.52°, p = 0.50; bimodal: −0.41°, p = 0.56), the overall systematic error for tactile responses revealed a small but significant leftward bias during both unimodal (−1.90°, p = 0.01) and bimodal stimulation (−1.31°, p = 0.02). One potential reason for this is that during task performance all of our participants indicated their responses with their right hand. An unanticipated consequence of this was that their right arm was more extended than their left, thus leading to a slightly asymmetric body posture (i.e., a slight rotation of the torso toward the left, cf. Fig. 1B); yet the observed systematic error is small compared to the spatial disparities between stimuli and thus had no noteworthy impact on the analyses and conclusions presented in the following.

In order to investigate the degree of cross-modal interactions, we analyzed responses to auditory and tactile cues that were presented at different disparities ranging from −36° to 36° at 12° intervals. We again computed the overall auditory and tactile systematic errors, but this time separately for each level of interstimulus disparity. Figure 2C illustrates the cross-modal biases for auditory and tactile responses at different disparities. Note that for tactile targets (right column), we observed a general offset toward the left side of the body, consistent with the earlier observation of an overall systematic error of −1.9° in the unimodal tactile condition. More importantly, we found that the responses to both modalities displayed a strong bias in the direction of the other cue and that the size of this bias increased almost perfectly linearly with stimulus disparity (r > 0.99, p < 0.001 for both cases).

Because our participants indicated the location of auditory and tactile cues in the bimodal condition with two subsequent responses, it is possible that the action of the first response and still having the first response visible on the screen may have lead to a tendency to place their second response closer to the location of the first response. As a result, there is the possibility that the observed bias patterns emerged from a “reference-related bias,” rather than from a perceptual interaction between the presented cues. To investigate this possibility, we repeated our analysis using only those trials in which the modality of interest was localized first. In these cases, there was no visual reference that could influence the participants’ responses, and any systematic bias had to be of a cross-modal nature (i.e. “perceptual bias”). Conversely, the relative contribution of the reference-related bias was obtained by subtracting the perceptual bias from the bias observed in trials where the respective modality was localized second. The perceptual biases (black bars, Fig. 2D) closely follow the pattern observed earlier (cf. Fig. 2C), namely that bimodal stimulation introduces a localization bias that linearly increases with stimulus disparity (r > 0.99, p < 0.001 in both the auditory and the tactile modality). However, the magnitude of bias is now decreased by about 15% to 30% depending on modality and disparity between the cues. The reference-related bias (gray bars) depends on stimulus disparity as well (auditory: r = 0.95, p = 0.001; tactile: r = 0.98, p < 0.001), but did not differ across modality (p > 0.05). The absence of the earlier observed leftward bias of responses to tactile cues supports the notion that this bias did not result from auditory-tactile interactions. In summary, the larger portion of the initially observed bias arose from perceptual interactions between the auditory and tactile modalities, with another smaller part that must be attributed to a reference-related bias that is only present during the second response. Therefore, all following behavioral analyses will be performed exclusively on first-response (and thus reference-bias free) data.

Now that we had extracted the purely cross-modal portion of response biases we investigated the latter in more detail. ANOVA testing revealed that the influence of auditory stimulation on tactile localization performance is stronger than vice versa (F(6, 78) = 6.54, p < 0.05): On average auditory localization performance is biased towards the tactile stimulus by about 17% of the angular distance between the cues. Tactile localization on the other hand is on average biased toward the auditory stimulus location by about 25% of the stimulus disparity. Note that there was no significant interaction between the effects of stimulus disparity and modality (F(6, 78) = 1.81, p = 0.11) thus indicating that apart from a difference in magnitude both modalities influenced each other in a similar fashion.

To assess the impact of similarity between the two stimuli on their mutual influence, we split the data between coherently and non-coherently amplitude modulated stimulus pairs (Fig. 2E). In this analysis, two-way repeated measures ANOVAs confirmed the dependency of bias size on stimulus disparity (auditory: F(6, 78) = 25.52, p < 0.001; tactile: F(6, 78) = 18.77, p < 0.001). In both modalities, the slope of the bias across disparities tended to be steeper for coherent stimulus combinations as compared to non-coherent ones. This suggests stronger cross-modal influences between more similar stimulus types. However, this could only be confirmed for the tactile modality, where we found a significant interaction between stimulus disparity and amplitude coherence (F(6, 78) = 3.22, p < 0.01). In the auditory modality, by contrast, the interaction between stimulus coherence and stimulus disparity did not reach significance (F(6, 78) = 1.93, p = 0.08). These results indicate that similarity between the presented cues primarily enhances the tactile localization bias, while the auditory localization bias, if at all, is affected to a much lesser extent. Finally, we were interested in whether the observed bias patterns were indeed caused by a gradual shift of the responses toward the other modality, as the histograms in Fig. 2C,E suggest, or whether they were caused by all-or-nothing integration on only a few trials. This would occur if on some trials both stimuli were localized at the same place, while in others the two modalities did not influence each other. As both possibilities potentially can explain the data, we repeated our initial analysis, this time excluding those trials in which the auditory and tactile cues were localized at the same direction, that is, within a maximum distance of 6° relative to each other, independent of the actual disparity level between the cues. The results are shown in Fig. 2F: Again, the magnitudes of bias are slightly decreased, but the overall patterns—including the linear bias increase across disparities (r = 0.99 and p < 0.001 in both modalities) and the overall leftward bias in the tactile modality—remain unchanged. These findings confirm that simultaneously presented auditory and tactile cues perceptually attract each other, even when they are not fully integrated.

### Integration in the auditory cortex

**Figure 3:**
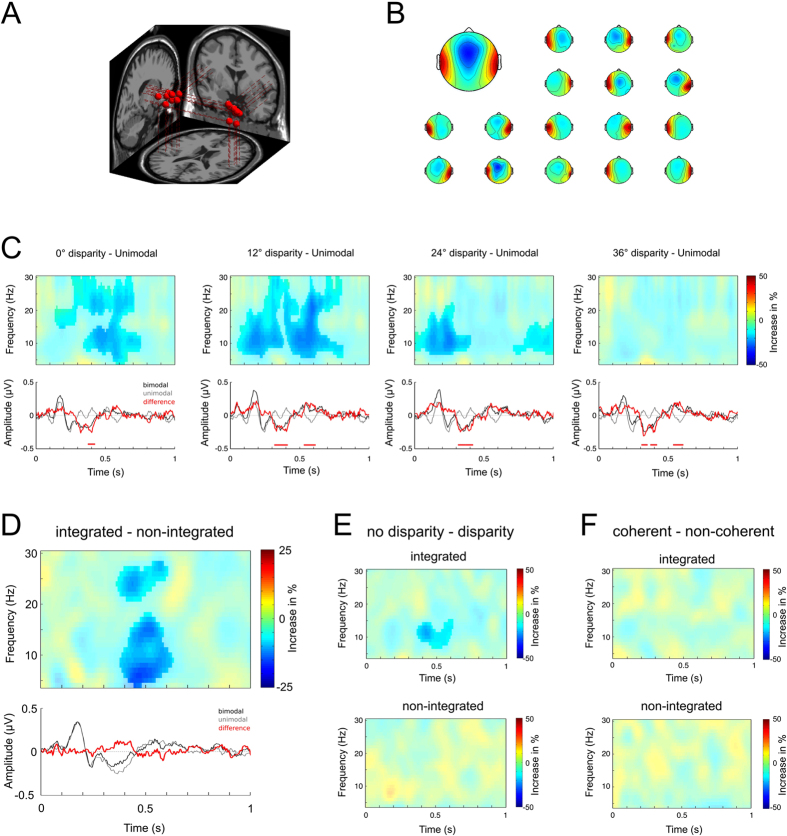
Audio–tactile integration in auditory cortex: (**A**) Dipole locations of independent components (IC) selected for further analysis and (**B**) their corresponding topographies, where the individual topographies are represented in the small icons, while the large one shows the average scalp projection over all ICs. (**C**) Upper row: Time-frequency representations of differences in temporal-cortical activity during unimodal and bimodal stimulation at different levels of stimulus disparity. Significant portions are shown in the original color; non-significant portions are color-reduced. Bottom row: The red traces show the differences between unimodal (gray traces) and bimodal (black traces) ERPs. Significant sections are indicated by red lines above the time axes. (**D**) Same conventions as in (**C**) but showing the differences between behaviorally integrated (i.e., both stimuli were localized within the same direction) and non-integrated (i.e., the stimuli were localized with an angular difference of 12° or more) trials. (**E**) Time-frequency representations of differences in neural activity during bimodal stimulation without (0°) and with (12°, 24°, or 36°) spatial disparity between the cues. The upper panel shows the case for trials in which the cues where behaviorally integrated, while the lower panel corresponds to trials in which the cues were not integrated. As before, significant portions are shown in the original color, while non-significant portions are color-reduced. (**F**) Same conventions as in (**E**) for the difference between coherent and non-coherent cue combinations.

At the cortical level, the integration of touch and sound has been shown to be related to neural activity in the auditory cortex^19^,^20^,^21^,^22^,^38^,^39^. Therefore we exclusively selected and analyzed those ICs from the EEG that were located within left or right auditory cortex and were activated by auditory stimulation. Across the pool of subjects we found a total of 16 ICs meeting these criteria. Their corresponding dipole locations and scalp projections are shown in Fig. 3A,B, respectively.

First, we tested if and how the spectral activities emerging from these ICs are altered in the presence of tactile cues. Therefore, we computed the time-frequency spectra for the different stimulus disparities in the bimodal condition and subsequently subtracted the ones obtained in the unimodal auditory condition. The results are shown in the top row of Fig. 3C (non-significant portions are color-reduced). At 0° disparity, bimodal stimulation caused a significant power decrease which was strongest in the alpha (8–14 Hz) and high beta (> = 20 Hz) range and most pronounced from 400 ms to 650 ms after stimulus onset. A similar pattern was observed at 12° disparity but with an additional alpha power decrease in the time range between 100 ms and 350 ms. At larger disparities, the alpha/beta cluster between 400 ms and 650 ms disappeared, while the earlier alpha cluster is reduced at 24° stimulus offset and disappears at 36° disparity. The finding that the alpha and beta power decreased in response to proximate stimulus combinations is stronger than for more distant ones cannot be explained by the mere presence of the tactile cue. Instead, the dependence of spectral patterns on stimulus disparity indicates that alpha and beta power desynchronization may be related to perceptual integration.

Previous studies have reported enhanced temporal-cortical ERP responses during bimodal audio-tactile stimulation as compared to the summed responses to the respective unimodal stimuli^19^,^24^,^27^. As alpha power has previously been linked to ERP activity^40^,^41^,^42^,^43^,^44^, we examined whether the observed time-frequency patterns indeed reflect an induced alpha/beta power reduction or merely the spectral equivalent of ERP differences in the time domain. The bottom row of Fig. 3C reveals that ERP differences (red traces) between bimodal (black) and unimodal auditory (gray) trials were very similar across all disparity levels. Furthermore, the overlap between the significant portions in the ERPs (p < 0.05, red lines above the time axes) and the observed spectral clusters is too inconsistent to suggest a meaningful relationship between the two. In conclusion, the presence and similarity of ERP differences across all disparity levels suggests that they reflect the general presence of the additional stimulus in the bimodal condition, rather than perceptual integration. We investigated this in more detail by repeating our ERP analysis, but this time using an additive model (i.e. comparing the responses during bimodal stimulation vs. the summed responses in the two unimodal conditions). In contrast to earlier reports^19^,^24^,^27^ the additive model did not reveal any significant differences between uni- and bimodal stimulation. This underlines the notion that the ERP differences we observed earlier may be attributed to the additional presence of the tactile stimulus, while the observed alpha and beta power desynchronization is more likely to reflect audio-tactile integration as such.

To address this matter more directly, we analyzed integration-related EEG activity based on the behavioral performance of our participants. Therefore, we divided the bimodal condition into perceptually integrated trials; that is, trials in which the participants localized auditory and tactile cues within the same direction (+/−6°) and nonintegrated trials where the responses were segregated by 12° or more. This was similar to the behavioral analyses reported earlier. Note that auditory–tactile stimulus combinations with smaller disparities were more likely to be integrated while larger disparities contributed more trials to the nonintegrated portion of the data. As a result, the comparison between integrated and nonintegrated trials may yield differences that were caused by stimulus disparity rather than by perceptual integration. To ensure that such secondary effects did not confound our analysis, we corrected for this by choosing the same number of integrated and nonintegrated trials for each level of stimulus disparity. Figure 3D shows the comparison of neural activity related to integrated versus non-integrated trials in auditory cortex. The time-frequency analysis (top panel) revealed two significant clusters between 400 ms and 600 ms after stimulus onset, one extending over the whole theta and alpha frequency range (5 Hz to 18 Hz, p = 0.014) and another one in the beta range (22 Hz to 27 Hz, p = 0.048). In both clusters, perceptual integration caused stronger desynchronization (i.e., a power decrease). Furthermore, because both alpha and beta frequencies negatively correlate with the BOLD signal^45^, these results are in line with earlier studies using fMRI^20^,^21^. When we analyzed the corresponding ERP traces, however, we found no significant differences between perceptually integrated and non-integrated trials (Fig. 3D bottom panel), thus confirming the earlier notion that audio–tactile integration in auditory cortex is reflected by induced oscillatory changes rather than by time-locked activity.

In view of both our own results regarding behavioral localization performance and the findings in earlier studies^4^,^6^, we were interested in whether neural activity related to integration of spatially congruent stimulus combinations (i.e., 0° stimulus disparity) differs from what occurs with the (inaccurate) integration of spatially incongruent stimulus combinations (±12° to ±36° stimulus disparity). Figure 3E shows the time-frequency representations of the respective comparison (i.e., no disparity–disparity), which we performed independently for integrated (top panel) and non-integrated (bottom panel) trials. Perceptually integrated trials display significantly stronger alpha power desynchronization in response to spatially congruent—as compared to spatially incongruent—stimulus combinations. The timing of this difference is consistent with the neural activity pattern that we found to be associated with behavioral integration (cf. Fig. 3D). Non-integrated trials, on the other hand, did not display any significant differences between spatially congruent and incongruent stimulation. It is important to note that, in both cases shown (integrated vs. non-integrated) the compilation is agnostic of actual disparity; only the subjects’ responses differed. In accordance with previous studies^27^ stimulus disparity had no effect on the ERP. These observations suggest that neural activity related to perceptual integration in auditory cortex is modulated by stimulus disparity even when the behavioral answers are indistinguishable.

Finally, we investigated whether the observed neural patterns are also modulated by stimulus coherence (i.e., depending on whether the amplitude profiles of auditory and tactile cues were matching or not), analogous to the effects observed in behavior. However, a direct comparison between coherent and non-coherent stimulus pairs did not reveal any significant differences with respect to ongoing oscillatory activity (Fig. 3F) or time-locked ERP responses. This indicates that coherence of bimodal stimuli influences the described desynchronization of alpha and beta activity only indirectly by modulating the probability of integration.

Summarizing, our overall findings indicate that perceptual and behavioral integration of audio–tactile cues is largely reflected by dynamic changes in induced oscillatory activity in the temporal cortex.

## Discussion

In the unimodal and the spatially congruent bimodal conditions, we found localization performance and error patterns associated with both auditory and tactile stimuli to be similar to the ones reported in previous experiments^17^,^18^. However, when disparities were introduced between the stimuli, the estimates for auditory and tactile cue locations were drawn toward the cue in the respective other modality. The size of this effect linearly increased with angular distance between the cues and was more pronounced for stimulus combinations with matching amplitude profiles. In order to focus on the main objectives of the present study, we did not present any angle- and error-specific interactions in detail, but overall our findings that both the spatial congruence and the similarity between the stimuli affect the likelihood of perceptual integration are in line with earlier studies using other modality combinations^3^,^4^,^6^,^7^,^46^,^47^. Further analysis confirmed that although the order of responses to tactile and auditory stimuli makes a minor contribution to the observed bias pattern, the largest part nevertheless arises from cross-modal interactions, which in turn were best explained by gradual rather than “all-or-nothing” integration of audio-tactile cue combinations.

Our principal findings regarding cross-modal effects on localization performance were also reflected in the EEG activity originating from temporal–cortical sources. ICs at these locations are most likely related to audio-cortical areas, since they – unlike ICs at more central and parietal locations for instance – are not easily confused with ICs related to other modalities, even if they are not entirely accurately localized within and across individual subjects. Comparing the impact of unimodal auditory and bimodal stimulation on the activity patterns at these sources revealed differences in alpha and beta power that cannot be explained by the presence of the additional tactile stimulus alone. More specifically, we found two spectral clusters, an early one centered around 100 ms to 300 ms after stimulus onset and a later one between approximately 400 ms and 600 ms. The first cluster became significant only at 12° and 24° stimulus disparity but not at 0°, which argues against a general involvement of the cluster in perceptual integration. Conversely, it also seems not to be related to spatial incongruency per se, as it also did not become significant at larger disparities (i.e., at 36°). Thus, a plausible explanation is that the observed differences are generated by ambiguous stimulation, namely in situations where perceptual inaccuracies make it difficult to judge whether both stimuli originate from the same or different locations. This would also be in line with the observation that it is strongest at 12° and then gradually decreases with stimulus disparity. The second cluster, on the other hand, becomes significant at 0° and 12° disparity and not at larger offsets (although residuals of the difference can be still seen in the spectral representation). This pattern is consistent with the likelihood of perceiving both stimuli as originating from the same location, thus suggesting an involvement in perceptual integration. This is also backed by the fact that alpha and beta activity negatively correlate with BOLD activity^45^, which is concordant with earlier reports of increased hemodynamic responses related to audio–tactile interactions in the auditory cortex^20^,^21^.

Next to increased BOLD responses, previous work also revealed enhanced ERPs in response to bimodal stimulation as compared to the combined activity of unimodal tactile and auditory stimulation. Typically this enhancement occurs at around 80–100 ms^19^,^24^,^27^ and subsequently at around 300–400 ms after stimulus onset^24^. ERP differences with a similar timing are also visible in our data but they only become significant at the later time interval (i.e., at 300–400 ms). Note, however, that our results were obtained by comparing bimodal and unimodal auditory stimulation, while most other studies^19^,^27^ used an additive model approach (i.e. comparing bimodal activity vs. the sum of unimodal auditory and tactile activity). When we performed the equivalent analysis on our data, we observed no significant ERP differences. This might be due to the fact that other studies used electrical stimulation of the median nerve and pure tones with sharp onsets (7.5 ms until maximum amplitude), while our stimuli were slowly amplitude modulated, reaching maximum amplitude only after about 150 ms at the earliest, and thus eliciting a much later and longer response. Hence these stimuli, which do not have a hard onset, may be not ideal for eliciting a strong time-locked ERP.

Importantly earlier studies^2^,^4^,^6^ as well as our own behavioral results indicate that cross-modal interactions can result in different levels of sensory integration. Full integration leads to perceiving both cues as corresponding to one single event and thus to emanate from the same source, while during partial or non-integration the stimuli are perceived as co-occurring but separate events^3^,^4^,^6^. As most experiments investigating temporal-cortical integration of touch and sound either relied on passive stimulation^19^,^21^,^38^ or only collected responses to one modality at a time^14^,^25^, the distinction between perceptual unification and more general interactions due to co-occurrence could not be made. By contrast, our participants responded to both modalities, which allowed us to identify trials in which both cues were – irrespective of their actual spatial disparity – localized in the same direction and thus likely to be fully integrated. We found that alpha desynchronization is significantly stronger for fully integrated as compared to non-integrated trials, thus demonstrating that neural activity in the auditory cortex plays an active role regarding the perceptual and behavioral outcome of audio–tactile stimulation. In this context it is important to note that a considerable portion of the trials that were classified as “non-integrated” may nevertheless show partial-integration in the form of perceptual biases as demonstrated in Fig. 2F. This again supports the hypothesis that full perceptual integration involves other (additional) neural mechanisms than general cross-modal interactions per se. Further evidence in this respect comes from the observation that ERPs were not systematically altered by the level of perceptual integration. Moreover, while the spatial disparity between integrated cues modulated the respective alpha desynchronization, it had no effect on the observed ERP patterns – neither in our data nor in the ones of previous studies^27^.

Finally, previous studies have demonstrated that the degree of perceptual similarity between stimuli may modulate the strength of cross-modal integration^3^,^4^,^5^,^6^,^7^,^16^,^48^. In line with these findings we observed that coherently modulated cues produced larger perceptual biases than non-coherent ones. Accordingly we expected these differences to be also reflected in integration-related alpha and/or beta activity. Contrary to our expectations, however, we were not able to find any effects of temporal stimulus coherence as such in our EEG data. One explanation for this discrepancy between our observations and earlier ones^21^ may be, that other studies varied temporal stimulus coherence in terms of presenting the stimuli either simultaneously or with varying degrees of temporal offset. In our case the manipulations were much more subtle, as they did not affect stimulus coincidence but only the co-modulation of amplitude profiles. As a result the perceptual difference may have been too small to be reflected in the EEG, at least with regard to the limited number of relevant ICs.

In summary, we demonstrated that simultaneously presented auditory and tactile cues give rise to cross-modal interactions that lead to localization biases in both modalities. Analyzing the corresponding EEG data based on our participants’ behavioral responses not only confirmed earlier reports of the involvement of the auditory cortex in audio–tactile integration. The results also indicate that ERP responses and changes in ongoing oscillatory activity may reflect different levels of cross-modal processing. While the former seem to be evoked by the general presence of the tactile cue, the latter appear to be directly related to the perceptual unification of audio-tactile events.

## Additional Information

**How to cite this article**: Plöchl, M. *et al.* Oscillatory activity in auditory cortex reflects the perceptual level of audio-tactile integration. *Sci. Rep.* **6**, 33693; doi: 10.1038/srep33693 (2016).
